# PDSRS-LD: Personalized Deep Learning-Based Sleep Recommendation System Using Lifelog Data

**DOI:** 10.3390/s25206292

**Published:** 2025-10-10

**Authors:** Ji-Hyeok Park, So-Hyun Park

**Affiliations:** Department of Software Science, Dankook University, Jukjeon Campus, 152 Jukjeon-ro, Suji-gu, Yongin-si 16890, Gyeonggi-do, Republic of Korea; pjh72230235@dankook.ac.kr

**Keywords:** sleep research, recommender system, life log, wearable device, deep learning

## Abstract

This study proposes a Personalized Deep Learning-Based Sleep Recommendation System Using Lifelog Data (PDSRS-LD). Traditional sleep research primarily relies on bio signals such as EEG and ECG recorded during sleep but often fails to sufficiently reflect the influence of daily activities on sleep quality. To address this limitation, we collect lifelog data such as stress levels, fatigue, and sleep satisfaction via wearable devices and use them to construct individual user profiles. Subsequently, real sleep data obtained from an AI-powered motion bed are incorporated for secondary training to enhance recommendation performance. PDSRS-LD considers comprehensive user data, including gender, age, and physical activity, to analyze the relationships among sleep quality, stress, and fatigue. Based on this analysis, the system provides personalized sleep improvement strategies. Experimental results demonstrate that the proposed system outperforms existing models in terms of F1 score and Average Precision (mAP). These results suggest that PDSRS-LD is effective for real-time, user-centric sleep management and holds significant potential for integration into future smart healthcare systems.

## 1. Introduction

Sleep is a fundamental factor in maintaining both physical and mental health, playing a vital role in physical recovery, memory enhancement, emotional regulation, and various other aspects of well-being [1,2,3]. However, in modern society, poor sleep quality has become increasingly common due to irregular lifestyles, elevated stress levels, and excessive use of digital devices. Consequently, a variety of technology-driven approaches have been developed to assess and improve sleep quality collectively known as sleep tech [4,5,6].

Sleep tech refers to a set of technologies that collect and analyze sleep-related data to enhance sleep quality. Initially, these technologies took the form of simple monitoring devices, but they have since evolved to include bio signal-based analysis tools, sleep environment control systems, and personalized sleep guidance systems. Since the mid-2010s, IoT-based sensor technologies and mobile devices have enabled more advanced sleep tracking [7]. Subsequently, EEG-based sleep analyzers [8], AI-powered motion beds [9,10], smart alarms, and sleep pattern recognition applications have emerged. For instance, wearable devices now provide functions such as sleep stage detection and sleep scoring, which help users improve their sleep habits.

Thus, sleep tech has evolved from simple monitoring to personalized solutions that provide quantitative analysis of an individual’s sleep state. The development of sleep tech has significantly influenced traditional sleep research based on physiological signals, spurring numerous follow-up studies. Traditional sleep studies using EEG (electroencephalography), ECG (electrocardiography), and PPG (photoplethysmography) sensors have yielded effective outcomes in sleep stage classification and the diagnosis of sleep disorders [11,12,13]. These approaches, grounded in precise physiological data, have proven effective in elucidating sleep structures in controlled environments. However, they fall short in reflecting participants’ everyday life patterns due to experimental constraints. Specifically, there are limitations in analyzing the relationship between external factors such as physical activity, stress levels, and fatigue and sleep quality.

To overcome these limitations, this study proposes to develop a Personalized Deep Learning-Based Sleep Recommendation System Using Lifelog Data (PDSRS-LD). First, lifelog data collected from wearable devices are used to evaluate users’ stress and fatigue levels and construct individual user profiles. Second, sleep data are gathered using an AI motion bed. Finally, secondary training is performed based on user feedback data to optimize personalized recommendations. Unlike traditional recommendation systems such as collaborative filtering (CF) and content-based filtering (CBF), which provide static suggestions after initial training, PDSRS-LD incorporates both primary recommendations and user feedback to adapt to individual sleep patterns. This enables the system to offer actionable guidance for improving sleep behavior, thereby enhancing the personalization and effectiveness of sleep management.

Although general sleep hygiene guidelines (e.g., maintaining regular bedtimes, exercising, limiting caffeine intake, and ensuring a quiet environment) are well established, their effectiveness varies greatly among individuals. Factors such as lifestyle, physiological characteristics, occupational demands, and environmental conditions often limit the applicability of generic advice. Therefore, a personalized recommendation system that leverages multimodal lifelog data, physiological signals, and user feedback can provide actionable strategies tailored to each individual’s unique context. By adaptively refining recommendations over time, such a system delivers practical value that conventional one-size-fits-all advice cannot achieve.

## 2. Related Work

### 2.1. Sleep Research

Recently, EEG-based bio signal analysis has been increasingly adopted as a refined methodology in sleep research. EEG (electroencephalography) is a widely used technique for quantitatively distinguishing sleep stages and measuring brain activity during each stage, particularly effective for identifying NREM (non-rapid eye movement) and REM (rapid eye movement) phases [14]. DeepSleepNet, a deep learning model for automatic sleep stage classification using raw single-channel EEG data, was proposed in [11]. The model integrates convolutional neural networks (CNNs) and bidirectional long short-term memory (bi-LSTM) layers to effectively learn both time-invariant features and sequential transition patterns between sleep stages, showing strong performance in sleep stage scoring on real-world polysomnographic datasets. In addition, there has been active research on diagnosing sleep disorders such as sleep apnea and insomnia by integrating multi-channel EEG with ECG (electrocardiography) and EMG (electromyography) [15,16,17]. Recent studies aim to extend these physiological signal-based analyses into real time sleep monitoring and smart healthcare applications by combining them with IoT sensors and wearable devices. While this bio signal centric approach is effective for analyzing sleep structures, it has limitations in reflecting external factors such as daily activities, stress levels, and fatigue that influence sleep quality.

### 2.2. Recommender System

Recommender systems have been actively studied since the late 1990s in the context of e-commerce and online content platforms [18,19,20]. Early research focused on collaborative filtering techniques based on explicit rating data between users and items, recommending new items by leveraging users’ historical behavior or the preferences of similar users. Representative examples include Amazon’s product recommendation algorithm and Netflix’s movie recommendation model. Later, content-based filtering (CBF) approaches were introduced, which recommend similar items based on the attributes (e.g., genre, keywords) of previously preferred items. While effective in capturing individual preferences, CBF methods often suffer from bias due to overemphasis on similar content [21]. To address this issue, hybrid filtering methods were proposed, combining collaborative filtering and content-based filtering to improve both recommendation accuracy and diversity [18,22,23,24].

In recent years, deep learning-based recommender systems have emerged, aiming to enhance prediction accuracy by incorporating not only traditional rating data but also unstructured data such as behavioral logs, text, images, and bio signals [25,26,27,28].

### 2.3. Lifelog Research

Lifelogging refers to the digital collection and recording of various user-generated data across daily life, including activities, environmental factors, and physiological information. With advancements in artificial intelligence and sensor technologies, lifelogging has gained increasing attention across multiple domains. Initially, it centered on simple biometric metrics such as step count, distance travel, and heart rate. However, more recent developments have enabled the collection of unstructured data including location, ambient sound, light levels, app usage, conversation logs, and images enabling more sophisticated analysis of user behavior [27,29,30].

Lifelog data have been widely utilized in applications such as healthcare, wellness, smart homes, personalized content recommendations, cognitive function monitoring, and workplace productivity analysis. Data collected via wearable devices and smartphones are typically structured as time-series and are useful for long-term behavior and health status analysis [31,32,33].

Sano et al. [34] proposed a model that predicts individual stress levels and emotional states using lifelog data collected from smartwatches and smartphone sensors. Similarly, Zhongqiu et al. (2016) [35] analyzed behavioral patterns such as location, call duration, and app usage to identify early indicators of social isolation and mental health risks. More recently, attempts have been made to semantically summarize daily routines or detect signs of health anomalies in real time using natural language processing and image recognition techniques.

Lifelog data, therefore, goes beyond conventional structured health records, offering dynamic and context-aware information. When combined with multivariate analysis methods based on machine learning and deep learning, they form a powerful foundation for predictive modeling and personalized services [36,37,38,39].

This study aims to extend the scope of previous research by moving beyond simple sleep data analysis to develop a system that predicts and improves sleep quality using lifelog data. It focuses on integrating deep learning-based analytical techniques with data collected from wearable devices to enable a more precise and personalized sleep recommendation system.

## 3. The Proposed PDSRS-LD

**Figure 1:**
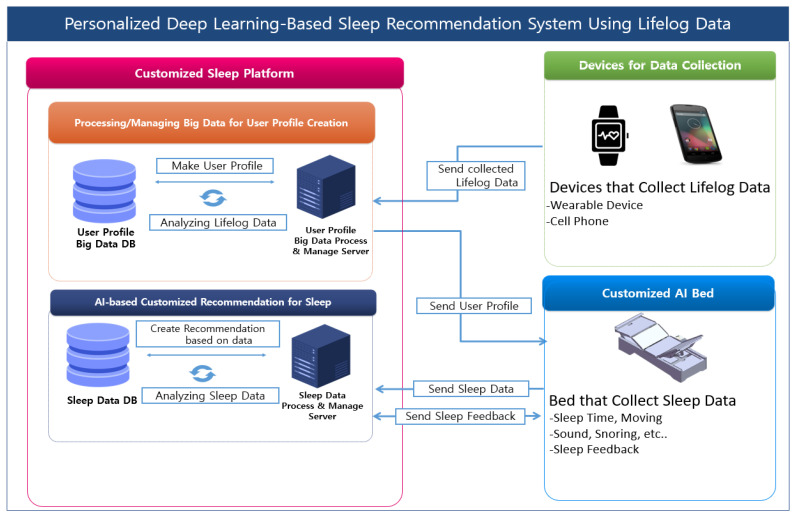
Personalized Deep Learning-Based Sleep Recommendation System Using Lifelog Data (PDSRS-LD).

The proposed PDSRS-LD in this study is illustrated in Figure 1. This platform is designed to improve sleep quality by accounting for users’ daily lifestyle patterns and consists of three key components: lifelog data, a customized AI motion bed system, and a big data driven sleep recommendation engine.

First, lifelog data are collected through wearable devices and smartphones that continuously monitor user’s daily activities and physiological signals. These data include information such as physical activity levels, sleep related behaviors, and biometric indicators. By analyzing variations in daily activity and stress indices, the system identifies factors affecting sleep quality and perform initial model training. This enables the platform to analyze correlations between lifestyle patterns and sleep quality.

Second, the customized AI motion bed system detects and analyzes physiological signals and body movements during sleep in real time. It monitors user’s posture changes, heart rate, and breathing patterns, contributing to the creation of an optimized sleep environment. This system includes a Central Control Board, which can automatically adjust environmental variables such as temperature and lighting, while also storing and transmitting sleep data in real time. Through this functionality, the system provides precise monitoring and facilitates the personalization of the user’s sleep environment.

Third, the big data–based sleep recommendation engine integrates the data collected from wearable devices and the AI bed to analyze sleep patterns and define individualized sleep conditions. It stores and manages long-term sleep-related data such as fatigue levels, stress indicators, and total sleep time. As user’ sleep data are continuously accumulated and analyzed, the system incrementally improves the precision of its personalized sleep recommendations.

Furthermore, the platform evaluates the accuracy of recommendations and incorporates user feedback to continually refine the AI recommendation engine. This feedback enables the delivery of improved, personalized sleep guidance. Through these components, the proposed PDSRS-LD platform builds an optimal sleep environment tailored to each user’s lifestyle and enhances the overall precision of personalized sleep recommendations.

Accordingly, this study aims to develop a personalized sleep recommendation system using AI-based analytical methods. By applying AI algorithms that consider each user’s fatigue and stress levels, and retraining the model with sleep data collected from the AI motion bed, the system can continuously improve the accuracy and personalization of sleep recommendations.

### 3.1. System Architecture of the PDSRS-LD

**Figure 2:**
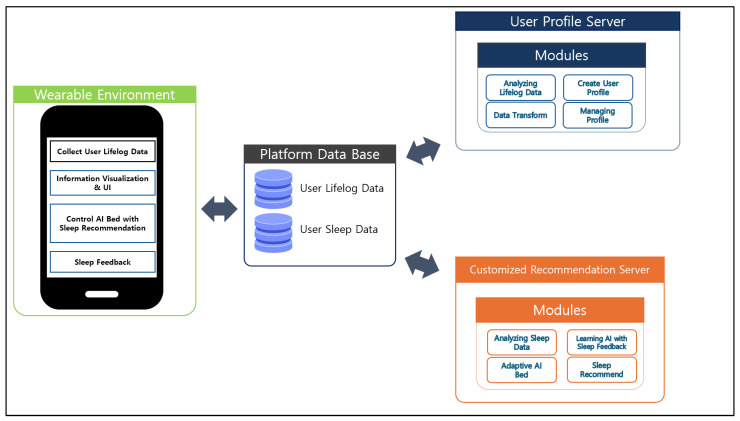
System Architecture of the PDSRS-LD.

Figure 2 visually illustrates the functional modules and data flow of the personalized AI-based big data sleep platform proposed in this study. The proposed system consists of four core components: Wearable Environment, Platform Database, User Profile Server, and Customized Recommendation Server.

First, the Wearable Environment module is responsible for facilitating interaction between the user and the system. It collects lifelog data from users in real time, provides visualization of sleep information, and offers a user interface (UI) for feedback and interaction. Based on the sleep recommendation results, it also controls the AI motion bed and collects user evaluation data through a post-sleep feedback mechanism.

In addition, the AI motion bed subsystem is described in greater detail in this study. A pressure-sensor grid was embedded under the mattress to capture posture and micro-movements, while load cells attached to the frame extracted respiratory and cardiac proxies. An inertial measurement unit (IMU) mounted on the frame was used for artifact detection, and environmental sensors (temperature, light, and noise) were placed bedside. The sampling rates were 10 Hz for the pressure sensors, 50 Hz for the load cells, 25 Hz for the IMU, and 1 Hz for the environmental sensors. All signals were resampled to 1 Hz and synchronized with wearable data using NTP (Network Time Protocol) timestamps, a widely adopted method for synchronizing system clocks across devices with millisecond-level accuracy. Pre-processing included Hampel filtering for artifact removal, band-pass filtering (0.1–0.5 Hz for respiration, 0.8–2.5 Hz for cardiac proxies), and posture smoothing with a hidden Markov model. After preprocessing, the bed-derived features were aligned with wearable features in identical nightly analysis windows, enabling feature-level early fusion in the recommendation model.

The collected data are stored in the Platform Database, which consists of two key data types: User Lifelog Data and User Sleep Data. This database serves as a central repository, and the stored data are used as input for subsequent analysis and recommendation modules.

The stored information is then transmitted to the User Profile Server, where lifelog data are analyzed, user profiles are created and managed, and data transformation processes are performed. This server constructs personalized sleep profiles based on users’ sleep habits and behavioral patterns.

Meanwhile, the analyzed sleep data and user profiles are transmitted to the Customized Recommendation Server, where advanced recommendation algorithms are executed. This module includes functions such as sleep data analysis, AI learning using sleep feedback, personalized sleep recommendations, and real time control of the AI bed through adaptive mechanisms.

Within this server, the internal sleep-analysis network is implemented as an early-fusion multilayer perceptron. It receives 68 input features per user-night, including wearable-derived indicators (steps, HR statistics, HRV-based stress and fatigue indices, sleep debt), AI-bed features (respiratory rate, posture transitions, micro-movement index, pressure entropy), and contextual variables (age, sex, circadian encoding of sleep timing, prior-night rating). The network consists of two hidden layers (128 units, ReLU, batch normalization, dropout = 0.2) followed by a 64-unit layer and two output heads: (a) a relevance head for ranking candidate sleep plans, and (b) an auxiliary regression head for next-day fatigue. The model is trained using Bayesian Personalized Ranking (BPR) loss combined with a Huber regression loss (λ = 0.1). Training uses AdamW (lr = 3 × 10^−4^, weight decay = 1 × 10^−2^), batch size = 256, max 100 epochs with early stopping, and results are reported as mean ± SD over three random seeds.

The proposed system realizes user centric sleep data collection, analysis, and personalized environment optimization through the organic linkage of these modules. It demonstrates the feasibility of an intelligent sleep recommendation platform aimed at improving sleep quality and promoting users’ overall health.

This modular architecture is specifically designed to support the research objective of delivering a personalized sleep environment through context-aware data analysis and real time feedback, centered on the individual user.

### 3.2. Construction of the User Profile

This study utilizes only the wearable sensor-derived subset of the real-world multimodal lifelog dataset presented by Chung et al. (2022) [40], which provides comprehensive human behavior data collected in naturalistic settings. The dataset consists of wearable sensor data collected over 12 weeks from 100 community-dwelling adult participants (50 in 2020 and 50 in 2024). Each participant was tracked continuously for 12 weeks, resulting in approximately 8400 user-nights of sleep and activity data. The participants were between 20 and 55 years of age, with a balanced gender distribution (52% female, 48% male). All participants were recruited as generally healthy adults; individuals with diagnosed sleep disorders, severe chronic illnesses, or psychiatric conditions were excluded. These criteria ensure that the data reflect typical daily behaviors and sleep patterns of healthy adults, while clarifying the scope and generalizability of the proposed architecture.

Among the multiple modalities in the original dataset, such as wearable sensor data, environmental sensor data, and mobile app usage logs, only the wearable device data, including step count, heart rate, stress level, fatigue index, and sleep-related metrics, were extracted and processed for this study.

All participants wore a Samsung Galaxy Watch Active2, equipped with a 3-axis accelerometer, gyroscope, photoplethysmography (PPG) heart rate sensor, and ambient light sensor. The watch continuously recorded step count and heart rate, while stress and fatigue indices were estimated using the Samsung Health algorithm based on heart rate variability (HRV). In this study, the HRV-based indices were not raw HRV metrics such as SDNN or RMSSD. Instead, they were proprietary indices provided by the Samsung Health algorithm, which computes aggregated stress and fatigue values from continuous HRV analysis using Galaxy Watch sensors. While the exact computational method is not publicly disclosed, these indices are widely employed in Samsung Health–based studies and were adopted here as representative HRV-derived features.

**Figure 3:**
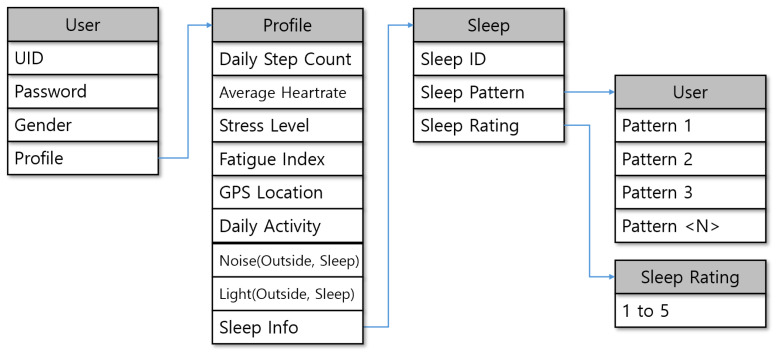
User Profile structure.

To precisely analyze individual sleep and behavioral characteristics and effectively apply them to a personalized sleep recommendation system, this study designed the User Profile structure illustrated in Figure 3. The profile integrates a user’s basic identification information, lifelog data, sleep-related information, and subjective evaluations of sleep quality. Specifically, it includes daily step count, average heart rate, stress level, fatigue index, GPS-based location data, daily activity, external noise, ambient light, and sleep-associated indicators. Each of these features was selected based on its established or hypothesized relationship with sleep quality. For example, step count and daily activity patterns are linked to circadian rhythm stability, while heart rate and HRV-derived stress and fatigue indices are widely used as physiological correlates of recovery and sleep depth. Ambient light and noise are known environmental disruptors of sleep, and GPS-based mobility data help to identify irregular routines or travel-related disruptions that negatively affect sleep quality. Including both physiological and contextual factors enables the system to construct a more comprehensive and personalized profile of the user. These data serve as important explanatory variables for analyzing the relationship between lifestyle and sleep patterns, and they are also utilized in clustering processes using algorithms such as K-means.

Sleep information is stored in a separate Sleep table. Each record contains a unique Sleep ID, an algorithm-derived Sleep Pattern, and a user-provided Sleep Rating. Users can submit multiple evaluations for different sleep patterns through a Sleep Rating table, which serves as a feedback-based personalization component in training the recommendation system. The sleep rating is provided on a scale from 1 to 5 and is a core factor in constructing a preference matrix between users and sleep patterns. In this context, the term “algorithm-derived Sleep Pattern” refers to nightly sleep characteristics automatically computed by the Samsung Health sleep algorithm in combination with our preprocessing pipeline. This information is generated automatically and is not manually entered by the user. In contrast, the sleep rating is provided directly by the participant on a 1–5 scale as subjective feedback after each night. Specifically, each record includes total sleep time, sleep onset latency, wake after sleep onset (WASO), and sleep efficiency, which together define the nightly sleep pattern stored in the Sleep table. In addition, participants were asked to provide a subjective evaluation of their sleep after each night. These evaluations were submitted on a 1–5 scale, reflecting the user’s perceived sleep quality. As a result, a single participant contributed multiple evaluations across nights, which served as feedback data for training the recommendation system.

All data underwent preprocessing to ensure compatibility with machine learning models. Numerical variables were normalized to maintain consistent scales, categorical variables were converted using one-hot encoding, and missing values were handled through mean imputation or removal. Specifically, missing values were relatively sparse (<2% of the dataset). For continuous variables such as step count, heart rate, and sleep duration, short gaps (<30 min within a day) were imputed using the mean of adjacent valid segments, while entire nights with missing wearable or bed signals were removed to avoid introducing noise. For categorical variables (e.g., activity labels), missing entries were coded as “unknown” and excluded from clustering. In total, 72 nights (≈0.9% of 8400 user-nights) were removed due to insufficient data quality, while the remaining missing values were imputed according to the above criteria.

The final User Profile is used as input to the recommendation system, functioning as a central component in various analytical modules, including clustering-based pattern classification, similar user identification, sleep recommendation algorithm training, and feedback-based personalized re-recommendation.

### 3.3. Data Selection Algorithm for Clustering

The core of lifelog-based sleep recommendation lies in effectively identifying and clustering physical activity, stress, and environmental factors that influence sleep quality. To achieve this, the present study applies an unsupervised learning approach using K-means clustering to classify characteristic daily patterns based on pre- and post-sleep data. The optimal number of clusters and combinations of features were validated through silhouette analysis.

**Figure 4:**
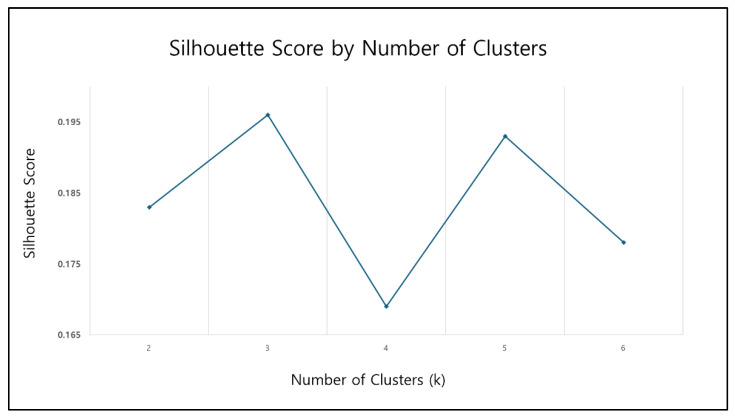
Initial clustering results with silhouette score.

In the initial experiments, clustering was performed using a total of eight indicators measured before and after sleep, including step count, mean and standard deviation of heart rate, light level, and noise level. As shown in Figure 4, the clustering result with k = 3 clusters yielded a silhouette score of 0.196, indicating relatively low clustering quality.

The clustering algorithm was refined through the following three step process:

1. Removal of Variables with Low Contribution to Clustering

Variables with high variance but limited impact on clustering such as day-noise, daylight, and sleep-light were excluded. The decision to remove such variables was based on correlation analysis, intra-cluster variance, and feature importance assessed via Principal Component Analysis (PCA). Specifically, features showing high correlation (|r| ≥ 0.90) or low ANOVA F-scores against provisional cluster labels were removed to reduce redundancy and improve separability.

2. Dimensionality Reduction Using PCA

To address multicollinearity and high dimensionality, PCA was applied to the refined feature set. The number of principal components was determined by retaining those that collectively explained at least 85% of the total variance, which resulted in three principal components. These components were then used as inputs for clustering to enhance pattern separation.

3. Algorithm Configuration and Selection

K-means clustering was performed in the reduced three-dimensional PCA space using the following configuration: Euclidean distance metric, init = ‘k-means++’, n_init = 50, max_iter = 500, tol = 1 × 10^−4^, and random_state = 42. The number of clusters (k) was determined by testing values from 2 to 6 and selecting the value with the highest silhouette score.

**Figure 5:**
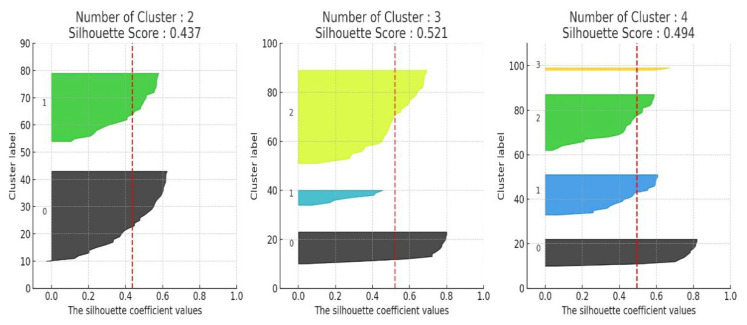
Clustering results after algorithm refinement, with improved silhouette score. Different colors indicate distinct clusters identified by the silhouette analysis (e.g., green = Cluster 1, black = Cluster 2, blue = Cluster 3, yellow = Cluster 4).

The results of applying K-means clustering to the improved feature set after dimensionality reduction are presented in Figure 5. With k = 3, the silhouette score improved significantly to approximately 0.52, indicating a clearer separation of sleep behavior patterns. Each cluster was profiled based on the number of users, average sleep quality score, and dominant lifestyle or physiological characteristics, as summarized in Table 1. This result suggests that the clustering method effectively distinguishes how daytime fatigue and stress levels impact sleep quality.

To enhance interpretability, Table 1 reports cluster means as representative values. The within-cluster variability was also examined: the standard deviation of sleep quality ranged from 0.4 to 0.7 across clusters, confirming that the average values are representative. Dominant characteristics were identified by comparing feature distributions and highlighting the most distinctive attributes of each group. Stress level was derived from HRV-based indices, independent of subjective sleep ratings, while activity patterns were categorized as follows: low activity (lowest quartile of daily step count), high activity (highest quartile), and irregular activity (high coefficient of variation in day-to-day step counts, above the 75th percentile).

Although the original dataset contained 100 participants, only 51 users are shown in Table 1. During the clustering process, users with ambiguous patterns or without a clear match to any cluster were excluded to ensure stable and interpretable cluster profiles. Thus, Table 1 summarizes the subset of 51 users with clearly assigned clusters.

The resulting clusters were subsequently used as input features for the hybrid recommendation algorithm, thereby enhancing the recommendation performance by incorporating the relationship between daytime activity and sleep behavior. As summarized in Table 1, Cluster 0 represents users with moderate stress levels and irregular activity patterns, Cluster 1 consists of users with high stress and low activity, and Cluster 2 includes users with low stress and high activity levels, which correspond to the highest average sleep quality among the groups. These profiles provide actionable insights for tailoring personalized sleep recommendations.

### 3.4. Cluster-Based Similar User Recommendation

Based on the clustering and silhouette validation results conducted in Section 3.3, each user is assigned to a specific cluster. When a sleep recommendation is requested, the system identifies users within the same cluster who exhibit similar patterns in selected features and who also demonstrate higher sleep quality scores than the target user. These matched users are then used as reference profiles for recommendation. In Algorithm 1, Step 2 retrieves users from the same cluster, and in practice this includes the filtering criterion described above. This step is presented in simplified pseudocode form, while the narrative text clarifies the actual implementation.

Through this process, the target user can identify potential issues in their daily lifestyle and evaluate whether the recommended sleep patterns are suitable for them. This evaluation contributes to the iterative refinement of a personalized user profile. This process is described in Algorithm 1 below.
**Algorithm 1: CBSUR (Cluster-Based Similar User Recommendation)**InputUserProfile, ClusterLabelsOutputSimilar User1.Let $C_{u}\;\leftarrow$ Cluster to which user u belongs2.Let S ← {v
∈ UserProfile | ClusterLabel [v] = $C_{u}$
∧
v
≠u}3.For each v
∈ S do:4.  Compute similarity using cosine similarity:5.  sim(u,v) = $\frac{\sum_{i=1}^{d}{x_{u,i}x}_{v,i}}{\sqrt{\sum_{i=1}^{d}x_{u,i}^{2}}\sqrt{\sum_{i=1}^{d}x_{v,i}^{2}}}\;$6.Let $v^{*}\leftarrow$ argument ${max}_{v\in S}sim$(u,v) 7.Return Similar User

The user with the highest similarity score within the same cluster is selected as the recommendation reference. The behavior and sleep patterns of this user are then presented as personalized suggestions.

This approach is characterized by its use of localized similarity within clusters, allowing the system to provide pattern-based recommendations grounded in real users who are most like the target individual.

### 3.5. Feedback-Based Personalized Re-Ranking

The user profile is primarily constructed from each subject’s own historical lifelog features and past sleep ratings, ensuring that recommendations remain individualized. To address data sparsity and cold-start issues, similarity with other users is incorporated only during the re-ranking stage. To reduce the subjectivity of the 1–5 sleep rating scale, multiple ratings across different nights are aggregated and normalized before being used. Moreover, clustering ensures that comparisons are restricted to users with broadly similar behavioral and physiological patterns, thereby mitigating the variability in individual satisfaction criteria.

In response to the initial recommendations, user feedback such as satisfaction levels or preferences is collected and transformed into a rating matrix that represents the user’s preferences across behavioral and sleep patterns. The second stage recommendation algorithm, referred to as Personalized Feedback-based Recommendation Algorithm (PFRA), utilizes this feedback data to compute personalized prediction scores.

The predicted rating for a user is calculated using the following formula:

$$\hat{R_{v,i}}=\frac{\sum_{v\in N(u)}sim(u,v)R_{v,i}}{\sum_{v\in N(u)}sim(u,v)}$$

Here, N(u) denotes the set of top-K users most like user u, where similarity is measured using metrics such as cosine similarity.

Algorithm 2 describes the procedure of this feedback-based re-ranking method.

The variables in the above equation are defined as follows. Let u denote the target user for whom the recommendation is generated, and v denote a neighboring user similar to u. The index i refers to a candidate sleep plan or lifestyle adjustment item that has not yet been rated by user u. $R_{v,i}$ denotes the rating that user v has assigned to item i on a 1–5 scale, reflecting the perceived sleep quality.
**Algorithm 2: PFRA (Personalized Feedback-based Recommendation Algorithm)**InputRatingMatrix R (user feedback)OutputTop K personalized recommendation list1.For each user u ∈R: 2.For each item i not rated by u:3.  Predict rating score $\hat{R_{v,i}}$ as:4.  $\hat{R_{v,i}}$ = $\frac{\sum_{v\in N(u)}sim(u,v)R_{v,i}}{\sum_{v\in N(u)}sim(u,v)}$5.  Where:6.  N(u): Top K similar users to u7.  sim(u,v): similarity between u and v8.Let Top$K_{u}\leftarrow$ Top K items ranked by $\hat{R_{v,i}}$ Return Top$K_{u}$


Based on the predicted scores, the system ranks potential recommendations and presents the top-K items most likely to match the user’s preferences. These items consist of actionable guidance derived from the sleep behaviors of similar high-quality sleepers, as well as lifestyle improvement strategies related to stress management, activity levels, and other daily habits. This approach aims to enhance recommendation accuracy and user satisfaction by directly incorporating user feedback into the personalized recommendation process.

The generated recommendations fall into two primary categories:

1. Sleep Schedule Adjustment—Suggestions for optimal bedtimes, wake-up times, and total sleep duration.
2. Lifestyle and Activity Optimization—Guidance on daily activity levels, exercise timing, and rest periods.

For example, when cumulative sleep debt is detected, the system advises the user to go to bed 30 min earlier than usual. If the daily step count is below the recommended threshold, it suggests engaging in a 20-min afternoon walk. When HRV-derived stress indices are high, it provides stress management or relaxation strategies. If ambient light or noise levels remain elevated at night, the system prompts the user to darken the room, reduce noise exposure, or adjust the bedroom temperature. These examples highlight that the recommendations are not generic but rather actionable and context-specific, tailored to each user’s behavioral, physiological, and environmental data.

In our implementation, Algorithm 2 should not be understood as a standalone module operating outside the deep learning framework. Instead, the feedback-based re-ranking logic is embedded into the overall architecture. A multilayer perceptron (MLP) jointly learns non-linear user–item interaction patterns from the lifelog feature space, while also integrating the re-ranking scores produced by Algorithm 2. The similarity-based scores act as an auxiliary signal, complementing the feature-level representation learning of the MLP. This hybrid design ensures that the final recommendation list reflects both personalized behavioral/physiological representations and collaborative feedback re-weighting, thereby improving robustness and adaptability.

## 4. Experiment and Evaluation

### 4.1. Impact of the Feedback-Based Re-Ranking Module

To quantitatively evaluate the performance of the proposed recommendation system, Mean Average Precision (mAP) and F1-score were employed as the primary evaluation metrics.

Mean Average Precision (mAP) is calculated by determining the precision of recommendation results for each user and then averaging these values. In this formula, *U* denotes the set of all users, $R_{u}$ is the set of relevant items for user *u*.

|$R_{u}|$ is the number of relevant items for user *u*, *P*@*k* is the precision at cutoff rank.

*k*, and $rel_{k}$ is an indicator variable that equals 1 if the item at position *k* is relevant and 0 otherwise.

It comprehensively reflects how well the system ranks relevant items near the top of the recommendation list. Specifically, mAP measures the proportion of relevant items contained within the top-K recommendations. A higher mAP value indicates that items likely to be preferred by the user are positioned closer to the top of the list.

F1-score is the harmonic means of precision and recall, offering a balanced assessment of the system’s overall performance. In the context of recommender systems, precision represents the proportion of recommended items that the user prefers, while recall refers to the proportion of all preferred items that were successfully recommended. By considering both metrics simultaneously, the F1-score provides a more balanced and comprehensive evaluation of recommendation quality. The detailed mathematical formulations of mAP and F1-score are summarized in Table 2.

For clarity, we also provide a toy example of mAP calculation. Suppose a user’s Top-5 recommendation list is [✔, ✘, ✔, ✘, ✔], where three items are relevant. The precision values at the relevant positions are 1.0, 0.667, and 0.6, giving an Average Precision (AP) of (1.0 + 0.667 + 0.6)/3 ≈ 0.756. The reported mAP@5 is the mean of such AP values across all users.

To avoid information leakage, we adopted a grouped split by user: 70% of users were assigned to the training set, 10% to validation, and 20% to test, with nights kept in chronological order within each user. Hyperparameters were tuned on the validation set, and final results were reported on the disjoint test set, averaged over three random seeds. The same protocol was applied to the REST baseline for a fair comparison.

To evaluate the contribution of the Personalized Feedback-based Recommendation Algorithm (PFRA) to the overall performance of the proposed PDSRS-LD system, we conducted an ablation study by removing the PFRA module from the recommendation pipeline. In this configuration, the system generated recommendations solely based on the initial clustering and similarity-based matching, without incorporating user feedback into the re-ranking process.

The comparison between the baseline PDSRS-LD (without PFRA) and the PFRA-enhanced version is presented in Table 3.

The results indicate that integrating PFRA improved the mAP by approximately 4.6% and the F1-score by 4.1% compared to the version without PFRA. These gains confirm that incorporating user feedback into the re-ranking stage enhances the personalization capability of the system, leading to more accurate and user-relevant sleep and lifestyle recommendations.

By directly integrating user feedback into the recommendation loop, the PFRA module refines the system’s outputs to better align with individual preferences, thereby improving recommendation accuracy.

### 4.2. Comparison with State-of-the-Art Sleep Analysis Model

To evaluate the proposed PDSRS-LD system against a state-of-the-art sleep analysis model, we considered REST, proposed by Duggal et al. in 2020 [41], which achieved outstanding performance in sleep stage classification tasks with an accuracy of 0.83 and an F1-score of 0.81 on the Sleep-EDF Expanded dataset.

For a fair comparison, we integrated REST as a preprocessing module for sleep stage classification, replacing the internal sleep analysis process in PDSRS-LD. The outputs of REST, namely the predicted sleep stages, were used as part of the input feature set for the recommendation stage, while keeping the PFRA re-ranking module unchanged. The performance comparison between the original PDSRS-LD and the REST-integrated variant is summarized in Table 4.

The results show that replacing PDSRS-LD’s internal sleep analysis module with REST’s outputs led to a performance decrease of 3.1% in mAP and 5.2% in F1-score. This suggests that PDSRS-LD’s internal design, which jointly optimizes lifestyle and sleep pattern features, is already well-adapted to the personalized recommendation task. While REST achieves strong results in general-purpose sleep stage classification, its integration did not yield additional benefits in the context of PDSRS-LD’s holistic recommendation pipeline, highlighting the efficiency and task-specific optimization of the proposed system.

### 4.3. Performance Comparison with Traditional Recommendation Approaches

As shown in Table 5, from the perspective of recommendation accuracy, the proposed PDSRS-LD model achieved the highest Mean Average Precision (mAP) score of 0.412, outperforming traditional models such as CF (0.378), CBF (0.341), and the baseline hybrid model (0.395). This result indicates that the top-ranked items in the recommendation list are more likely to match the user’s actual preferences, demonstrating superior recommendation quality.

In terms of the balance between precision and recall, as measured by the F1-score, the proposed model also recorded the highest value at 0.563. This implies that the recommendation system not only delivers accurate suggestions but also effectively captures a wide range of user relevant items without omitting key preferences.

**Figure 6:**
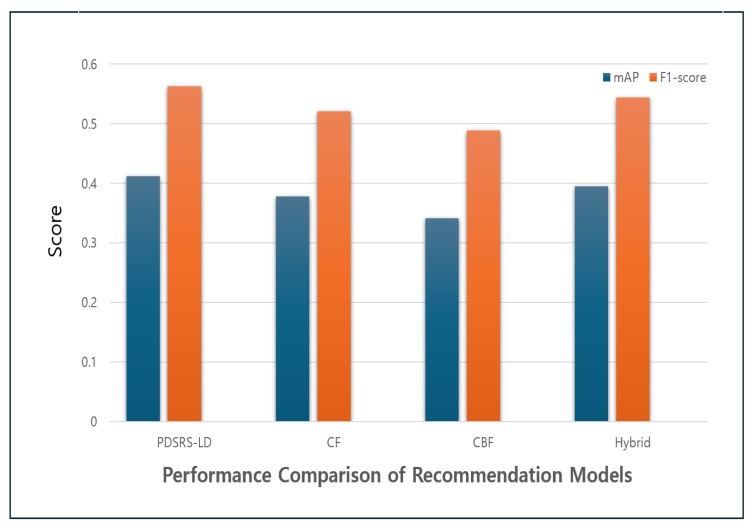
Performance Comparison of Recommendation Models.

Figure 6 shows the performance comparison of recommendation models. Although the baseline hybrid model showed slight improvements over the individual CF and CBF models, it still underperformed compared to the proposed PDSRS-LD. Although the baseline hybrid model showed slight improvements over the individual CF and CBF models, it still underperformed compared to the proposed PDSRS-LD. This finding suggests that merely combining simple algorithms may have limited effect, underscoring the importance of incorporating domain specific features such as user behaviors and sleep patterns into the model design.

## 5. Conclusions and Future Works

Traditional sleep recommendation systems have generally relied on rule-based or survey-driven methods, providing generic advice such as “go to bed earlier” or “reduce caffeine intake,” without adapting to individual differences. More advanced systems monitored physiological signals (e.g., EEG, ECG, PPG) to classify sleep stages or detect disorders, but their recommendations remained static and limited to laboratory conditions. Furthermore, general recommender approaches such as collaborative filtering (CF) and content-based filtering (CBF) rely on static recommendation structures that cannot adapt to changes in users’ conditions or preferences over time. These limitations highlight the need for a more sophisticated, user-centered model design that considers the dynamic interplay of factors such as stress, fatigue, physical activity, ambient light, and noise.

To address these issues, this study designed and implemented a personalized deep learning–based sleep recommendation system (PDSRS-LD) that integrates multimodal lifelog data, AI motion bed signals, and user feedback. By leveraging information from daily life—such as activity levels, ambient light and noise, heart rate, and sleep evaluations—the system effectively analyzes sleep patterns and delivers individualized, adaptive, and context-specific recommendations.

The proposed model demonstrated superior performance compared to traditional CF, CBF, and hybrid (CF + CBF) methods, achieving higher values in both mean average precision (0.412) and F1 score (0.563). These results suggest that the model, which comprehensively learns from user’s lifelog data and applies personalized recommendation strategies, is more effective at predicting and supporting actual sleep behavior than conventional approaches. In this study, subjective ratings on a 1–5 scale were used as feedback to personalize recommendations. While effective in capturing user preferences, such ratings are inherently subjective and may vary across individuals. Objective sleep scoring approaches, such as EEG-based staging, HRV-derived sleep indices, or wearable-provided sleep scores, could offer more standardized and reliable measures of sleep quality. These data, however, were not available in the present dataset, and our design focused on developing a system deployable in everyday settings using widely available wearable and environmental signals. Future work will incorporate objective sleep scoring to complement subjective ratings, thereby enhancing the robustness and generalizability of the system. Another design choice was that nightly sleep outcomes (e.g., total sleep time, sleep onset latency, WASO, and sleep efficiency) were not included as clustering features. These variables were intentionally treated as outcome measures rather than inputs, in order to avoid circularity and to preserve interpretability when examining how lifestyle-based clusters map onto sleep results. Nevertheless, incorporating such sleep outcomes into a hybrid clustering framework could be a promising future extension, enabling more nuanced profiling of the interaction between daily behaviors and sleep quality.

However, this study has certain limitations. It does not consider medical information such as chronic conditions or acute illnesses that may significantly affect sleep. Therefore, for users with specific or atypical health conditions, the accuracy of the recommendations may be limited. Future work will focus on integrating comprehensive medical data including chronic and acute illness histories, sleep disorder diagnoses, cardiovascular conditions, and mental health records into the recommendation framework. This enhancement aims to improve the model’s adaptability and personalization for specific health populations whose sleep quality may be significantly influenced by medical factors. Furthermore, real-time health status monitoring combined with an attention mechanism could refine recommendations in response to dynamic physiological changes. Beyond sleep strategies, the proposed system could evolve into a holistic health management platform that also monitors daily stress levels and suggests tailored physical and leisure activities, thereby supporting both preventive healthcare and individualized lifestyle optimization.

In addition, several technical and data-related limitations should be acknowledged. First, the dataset was limited to 100 participants, which may not fully capture demographic or lifestyle diversity. Second, wearable-derived signals are prone to noise, missing values, and device-specific variability, which may affect the reliability of extracted features. Third, the use of subjective sleep ratings on a 1–5 scale introduces inter-individual variability, even though normalization was applied across nights. Fourth, the current framework primarily reflects short-term behavioral feedback and has limited ability to model long-term adaptation. Finally, the system has not yet been validated in clinical populations, and its current applicability remains within wellness and lifestyle contexts rather than medical diagnostics. Addressing these issues in future work will be essential to further enhance the robustness, generalizability, and clinical applicability of the proposed system.

## Figures and Tables

**Table 1:** Cluster profiles derived from silhouette analysis results (k = 3).

Cluster Label	Number of Users	Average Sleep Quality	Dominant Characteristics
0	10	3.9	Moderate stress irregular activity
1	4	2.8	High stress low activity
2	37	4.4	Low stress, high activity

**Table 2:** Formulas of mAP and F1-score evaluation criteria.

Evaluation Criterion	Formula	Functional
mAP	$mAP\;=\;\left(\frac{1}{\left|U\right|}\right)\times \;\sum_{\left\{u\in U\right\}}\left(\frac{1}{\left|R_{u}\right|}\right)$×$\;\sum_{k=1}^{\left|R_{u}\right|}P@k\;$$\times \;rel_{k}$	Measures how well relevant items are ranked higher in the recommendation list
F1-score	$F1-\mathrm{score}=2\cdot \frac{Precision\cdot Recall}{Precision+Recall}$	Balances precision and recall evaluating overall recommendation effectiveness

**Table 3:** Performance comparison with and without the PFRA module.

Model	mAP	F1-Score
Proposed PDSRS-LD (except PFRA)	0.394	0.541
Proposed PDSRS-LD (include PFRA)	0.412	0.563

**Table 4:** Performance comparison between original PDSRS-LD and REST-integrated variant.

Model	mAP	F1-Score
Proposed PDSRS-LD (original)	0.412	0.541
Proposed PDSRS-LD (Sleep stage input)	0.399	0.513

**Table 5:** Comparison of Performance Results.

Model	mAP	F1-Score
Proposed PDSRS-LD	0.412	0.563
CF (collaborative Filtering)	0.378	0.521
CBF (Content-Based Filtering)	0.341	0.489
Hybrid (CF + CBF)	0.395	0.544

## Data Availability

The data presented in this study is available on request from the corresponding author.

## References

[1] Ramar K., Malhotra R.K., Carden K.A., Martin J.L., Abbasi-Feinberg F., Aurora R.N., Kapur V.K., Olson E.J., Rosen C.L., Rowley J.A. (2021). Sleep is essential to health: An American Academy of Sleep Medicine position statement. J. Clin. Sleep Med..

[2] Sambou M.L., Zhao X., Hong T., Fan J., Basnet T.B., Zhu M., Wang C., Hang D., Jiang Y., Dai J. (2021). Associations between sleep quality and health span: A prospective cohort study based on 328,850 UK biobank participants. Front. Genet..

[3] Okano K., Kaczmarzyk J.R., Dave N., Gabrieli J.D., Grossman J.C. (2019). Sleep quality, duration, and consistency are associated with better academic performance in college students. npj Sci. Learn..

[4] Mathunjwa B.M., Kor R.Y.J., Ngarnkuekool W., Hsu Y.-L. (2025). A Comprehensive Review of Home Sleep Monitoring Technologies: Smartphone Apps, Smartwatches, and Smart Mattresses. Sensors.

[5] Aziz S., A M Ali A., Aslam H., A Abd-alrazaq A., AlSaad R., Alajlani M., Ahmad R., Khalil L., Ahmed A., Sheikh J. (2025). Wearable Artificial Intelligence for Sleep Disorders: Scoping Review. J. Med. Internet Res..

[6] Birrer V., Elgendi M., Lambercy O., Menon C. (2024). Evaluating reliability in wearable devices for sleep staging. npj Digit. Med..

[7] Surantha N., Kusuma G.P., Isa S.M. Internet of things for sleep quality monitoring system: A survey. Proceedings of the 2016 11th International Conference on Knowledge, Information and Creativity Support Systems (KICSS).

[8] Feinberg I., Koresko R.L., Heller N. (1967). EEG sleep patterns as a function of normal and pathological aging in man. J. Psychiatr. Res..

[9] Liu Q., Kenny M., Nilforooshan R., Barnaghi P. (2021). An intelligent bed sensor system for non-contact respiratory rate monitoring. arXiv.

[10] Papillon O., Goubran R., Green J., Larivière-Chartier J., Higginson C., Knoefel F., Robillard R. (2025). Sleep Position Classification using Transfer Learning for Bed-based Pressure Sensors. arXiv.

[11] Supratak A., Dong H., Wu C., Guo Y. (2017). DeepSleepNet: A model for automatic sleep stage scoring based on raw single-channel EEG. IEEE Trans. Neural Syst. Rehabil. Eng..

[12] Sun H., Ganglberger W., Panneerselvam E., Leone M.J., Quadri S.A., Goparaju B., Tesh R.A., Akeju O., Thomas R.J., Westover M.B. (2020). Sleep staging from electrocardiography and respiration with deep learning. Sleep.

[13] Korkalainen H., Aakko J., Duce B., Kainulainen S., Leino A., Nikkonen S., Afara I.O., Myllymaa S., Töyräs J., Leppänen T. (2020). Deep learning enables sleep staging from photoplethysmogram for patients with suspected sleep apnea. Sleep.

[14] Vyazovskiy V.V., Delogu A. (2014). NREM and REM sleep: Complementary roles in recovery after wakefulness. Neuroscientist.

[15] Cheng Y.-H., Lech M., Wilkinson R.H. (2023). Simultaneous Sleep Stage and Sleep Disorder Detection from Multimodal Sensors Using Deep Learning. Sensors.

[16] Chambon S., Galtier M.N., Arnal P.J., Wainrib G., Gramfort A. (2018). A deep learning architecture for temporal sleep stage classification using multivariate and multimodal time series. IEEE Trans. Neural Syst. Rehabil. Eng..

[17] Moridani M.K., Heydar M., Behnam S.S.J. A reliable algorithm based on combination of EMG, ECG and EEG signals for sleep apnea detection:(a reliable algorithm for sleep apnea detection). Proceedings of the 2019 5th Conference on Knowledge Based Engineering and Innovation (KBEI).

[18] Dong Z., Wang Z., Xu J., Tang R., Wen J. (2022). A brief history of recommender systems. arXiv.

[19] Wang H., Wang N., Yeung D.-Y. Collaborative deep learning for recommender systems. Proceedings of the 21th ACM SIGKDD International Conference on Knowledge Discovery and Data Mining.

[20] He X., Liao L., Zhang H., Nie L., Hu X., Chua T.-S. Neural collaborative filtering. Proceedings of the 26th International Conference on World Wide Web.

[21] Nguyen T.T., Hui P.-M., Harper F.M., Terveen L., Konstan J.A. Exploring the filter bubble: The effect of using recommender systems on content diversity. Proceedings of the 23rd International Conference on World Wide Web.

[22] Zhang S., Liu K., Yu Z., Feng B., Ou Z. (2023). Hybrid recommendation system combining collaborative filtering and content-based recommendation with keyword extraction. Appl. Comput. Eng..

[23] Shrestha J., Uddin M.N., Jo G.-S. (2008). Combining Collaborative, Diversity and Content Based Filtering for Recommendation System. J. Intell. Inf. Syst..

[24] Xiao Y., Zhong R. (2019). A hybrid recommendation algorithm based on weighted stochastic block model. arXiv.

[25] Ren X., Wei W., Xia L., Su L., Cheng S., Wang J., Yin D., Huang C. Representation learning with large language models for recommendation. Proceedings of the ACM Web Conference 2024.

[26] Tian J., Wang Z., Zhao J., Ding Z. Mmrec: Llm based multi-modal recommender system. Proceedings of the 2024 19th International Workshop on Semantic and Social Media Adaptation & Personalization (SMAP).

[27] Xia L., Huang C., Xu Y., Dai P., Bo L. (2022). Multi-behavior graph neural networks for recommender system. IEEE Trans. Neural Netw. Learn. Syst..

[28] Park J.-H., Lee J.-D. (2023). A Customized Deep Sleep Recommender System Using Hybrid Deep Learning. Sensors.

[29] Chen J., Li D., Bi K. (2025). LifeIR at the NTCIR-18 Lifelog-6 Task. arXiv.

[30] Tran L.-D., Gurrin C., Smeaton A.F. (2024). Lifelogging as An Extreme Form of Personal Information Management—What Lessons To Learn. arXiv.

[31] Hong J., Seol Y., Lee S., Yoon J., Lee J., Park K.-S., Ha J.-W. (2024). Prediction of Cognitive Impairment Using Sleep Lifelog Data and LSTM Model. Mathematics.

[32] Yamada Y., Shinakwa K., Kobayashi M., Nemoto M., Ota M., Nemoto K., Arai T. Smartwatch-derived Acoustic Markers for Deficits in Cognitively Relevant Everyday Functioning. Proceedings of the 2023 IEEE International Conference on Digital Health (ICDH).

[33] Sakal C., Li T., Li J., Li X. (2023). Assessing cognitive function among older adults using machine learning and wearable device data: A feasibility study. arXiv.

[34] Sano A., Taylor S., McHill A.W., Phillips A.J., Barger L.K., Klerman E., Picard R. (2018). Identifying objective physiological markers and modifiable behaviors for self-reported stress and mental health status using wearable sensors and mobile phones: Observational study. J. Med. Internet Res..

[35] Li Z., Shi D., Wang F., Liu F. Loneliness recognition based on mobile phone data. Proceedings of the 2016 International Symposium on Advances in Electrical, Electronics and Computer Engineering.

[36] Palbar T., Kesavulu M., Gurrin C., Verbruggen R. Prediction of Blood Glucose Using Contextual LifeLog Data. Proceedings of the International Conference on Multimedia Modeling.

[37] Ni J., Chen B., Allinson N.M., Ye X. (2020). A hybrid model for predicting human physical activity status from lifelogging data. Eur. J. Oper. Res..

[38] Lee J.-Y., Lee S.Y. (2024). Development of an AI-Based Predictive Algorithm for Early Diagnosis of High-Risk Dementia Groups among the Elderly: Utilizing Health Lifelog Data. Healthcare.

[39] Kim K., Jang J., Park H., Jeong J., Shin D., Shin D. (2023). Detecting abnormal behaviors in dementia patients using lifelog data: A machine learning approach. Information.

[40] Chung S., Jeong C.Y., Lim J.M., Lim J., Noh K.J., Kim G., Jeong H. (2022). Real-world multimodal lifelog dataset for human behavior study. ETRI J..

[41] Duggal R., Freitas S., Xiao C., Chau D.H., Sun J. Rest: Robust and efficient neural networks for sleep monitoring in the wild. Proceedings of the Web Conference 2020.

